# Morphometric and radiological analysis of bicipital groove in adults for shoulder surgery

**DOI:** 10.6026/973206300220935

**Published:** 2026-02-28

**Authors:** Mayuri V Ghorpade, Manjusha K Tabhane, Deepali P Onkar, Varsha Bande

**Affiliations:** 1 Department of Anatomy, N K P Salve Institute of Medical Sciences, Nagpur, Maharashtra, India; 2 Department of Anatomy, Vedantaa Institute of Medical Sciences, Dahanu, Maharashtra, India

**Keywords:** Bicipital groove, intertubercular sulcus, proximal humerus, shoulder instability, morphometry, orthopedic surgery

## Abstract

Bicipital groove variations contribute to biceps tendon pathology, yet morphometric-radiological correlations remain underexplored. Therefore, it is of interest to measure length, width, depth and medial wall angle in 160 adult proximal humeri (80 dry bones, 80 CT scans). Groove depth positively correlated with medial wall angle (p<0.001); 18% showed shallow groove (<4 mm) predisposing to instability, with sexual dimorphism across dimensions. Shallow sulcus and reduced medial wall angle emerged as key subluxation risk factors. Findings advance preoperative imaging protocols for shoulder arthroplasty and cuff repair by establishing precise morphometric thresholds.

## Background:

The bicipital groove anatomically referred to as the intertubercular sulcus is a separate indentation on the anterior surface of the proximal humerus. It separates the greater and lesser tuberosities and provides a key conduit of the long head of the biceps (LHB) tendon as it passes, intra-articularly, to insert onto the supraglenoid tubercle [1]. The osseous structure of this groove and the transverse humeral ligament and the coracohumeral ligament create a fibro-osseous tunnel, which stabilizes the tendon during the complex biomechanics involved in shoulder abduction and rotation. Disorders of the LHB tendon are a common cause of anterior shoulder pain and are often, though not always, associated with rotator cuff pathology. The stability of the tendon in the groove depends strongly upon the depth of the sulcus and the angulation of its walls and in particular the medial wall [2]. A shallow groove or a supratubercular ridge may cause mechanical irritation to the tendon thereby tenosynovitis, fraying or dislocating of the tendon from the groove [3]. Consequently, knowledge of the detailed morphometry of this region is not an exercise in pure anatomy, but a clinical necessity of the orthopedic surgeons. Furthermore, proximal humerus is one of the common sites of surgical procedures, such as open reduction and internal fixation (ORIF) of fractures and shoulder arthroplasty. In hemiarthroplasty or total shoulder replacement surgery, the bicipital groove is an important landmark in determining how the humeral component is retroverted [4]. Inaccurate restoration of this anatomy can be a source of prosthetic failure, range of motion limitation and ongoing pain. Despite this, there is a wide amount of variability in the reported dimensions for the groove in different populations and reliance on outdated or non-population- specific data can cause surgical errors [5]. While traditional anatomical studies have used dry bone measurements, the arrival of high resolution Computed Tomography (CT) means that these parameters can be assessed in living subjects, providing a more clinically applicable set of measurements. However, few studies have been conducted simultaneously to analyze and compare morphometric data obtained from dry bones and radiological data to validate the reliability of the imaging metrics [6]. Therefore, it is of interest to report a complete morphometric and radiological analysis of the bicipital groove in an adult population and establishing the normal ranges of length, width, depth and medial wall angle and the prevalence of anatomical variations.

## Materials and Methods:

## Study design and setting:

This descriptive, cross-sectional observational study was done at the Department of Anatomy and Department of Radiology of a tertiary teaching hospital. Study was conducted for 24 months. The protocol was reviewed and approved by the Institutional Ethical Committee.

## Sample size and grouping:

There were a total of 160 samples in the study, split into two groups:

Group A (Osteological): 80 adult dry human humeri (40 right, 40 left) from the departmental bone bank.

Group B (Radiological): 80 shoulder CT scans (40 male, 40 female) retrospectively collected from the data archive (PACS) of the hospital.

## Inclusion and exclusion criteria:

## Inclusion criteria:

For Group A, Adult humeri fully ossified and without damage. For Group B, CT scans of adults (aged 18-65 years) for non-traumatic indications (e.g. vague shoulder pain) or the contralateral healthy shoulders in a trauma case.

## Exclusion criteria:

Specimens or scan that revealed: fractures of the proximal humerus, evidence of tumors, infectious pathology (osteomyelitis), severe osteoarthritis causing deformity of bony landmarks, history of previous shoulder surgery.

## Data collection instruments and methods:

## Group A (Dry Bones):

Measurements were made with a digital Vernier caliper (precision 0.01 mm) and a standard goniometer.

Length of the groove: Measured from the distal indistinct end of the groove to the transverse homeral ligament attachment site.

Width: Maximum distance between the crests of the greater and lesser tuberosities.

Depth: A thin ruler made of metal was placed across the tuberosities to bridge the groove. The depth value was measured from the ruler to the deepest point of the floor with the depth probe of the caliper.

Medial wall angle (MWA): The angle of the medial wall of the groove to the floor of the groove viewed from the midpoint of the sulcus length.

## Group B (Radiological):

CT scans were analyzed with DICOM viewer software with capabilities for multi-planar reconstruction (MPR). Axial sections at level of bicipital groove were selected.

Width: Measured as the distance between two tubercles on the axial slice.

Depth: A virtual line was drawn between the highest points of the greater and lesser tuberosities. A perpendicular line was dropped to the deepest part of the sulcus.

Medial wall angle: Is measured electronically using the angle tool on the axial.

## Statistical analysis:

Data were collected in Microsoft Excel and analysed in the software, SPSS version 25.0. Results were calculated as descriptive statistics (Mean ± Standard Deviation) of all the variables. Unpaired Student's t-test was used to compare means between sides (Right vs. Left) and Sexes (Male vs. Female). Pearson's correlation coefficient (r) was used to test the correlation between groove depth and medial wall angle. A p-value of <0.05 was considered as statistically significant.

## Results:

The study analyzed 80 dry humeri and 80 CT scans from adult specimens (estimated mean age 52.4 years for radiological group). Dry bone morphometry (Group A) revealed mean bicipital groove length of 82.45 mm (right 83.10 ± 5.05 mm vs left 81.80 ± 5.18 mm, p=0.245), width of 11.23 mm (right 11.65 ± 1.75 mm vs left 10.81 ± 1.88 mm, p=0.042), and depth of 4.65 mm (right 4.85 ± 1.15 mm vs left 4.45 ± 1.22 mm, p=0.134), with right humeri showing significantly larger width (p=0.042) and medial wall angles (58.4°C ± 6.2°C vs 54.1°C ± 5.8°C, p=0.003) (Table 1). CT measurements (Group B) demonstrated marked sexual dimorphism, with males exhibiting wider grooves (12.10 ± 1.65 mm vs 10.25 ± 1.45 mm, p<0.001), greater depth (5.12 ± 1.05 mm vs 4.15 ± 0.95 mm, p<0.001), steeper medial wall angles (57.2°C ± 5.9°C vs 52.8°C ± 6.1°C, p=0.001), and higher supratubercular ridge prevalence (17.5% vs 10.0%, p=0.329; overall 13.8%) (Table 2). Combined analysis showed strong positive correlation between groove depth and medial wall angle (r=0.78, p<0.001): shallow grooves (<4.0 mm) averaged 41.5°C ± 4.2°C (n=29), medium (4.0-6.0 mm) 56.2°C ± 5.1°C (n=102), and deep (>6.0 mm) 65.8°C ± 6.5°C (n=29), suggesting steeper walls in deep grooves enhance tendon stability (Table 3).

## Discussion:

The morphology of the bicipital groove is an important determinant of the pathology of the long head of the biceps tendon and is a pivotal factor in the success of shoulder reconstructive surgeries. This study combined dry bone morphometry and radiological evaluation to give a full profile on the intertubercular sulcus in an adult population. The results proved that the mean groove depth was about 4.65 mm in dry bones and 4.63 mm (combined average) in radiological scans. These findings are consistent with previous literature which broadly gives the definition of normal depth between 4 mm and 5 mm [7]. However, the very important result of this study is the strong positive correlation between the depth of the groove and the medial wall angle (r=0.78). The medial wall forms the chief buttress against medial subluxation of the tendon in external rotation of the shoulder. A shallow groove (< 4mm) coupled with a low medial wall angle (< 45 degrees) creates a "slide" instead of a "wall" and the risk of instability is greatly increased. Our data implies that about 18% of the population has this high risk morphology. This is associated in the clinical experience of patients with chronic anterior shoulder pain who often show shallow grooves on imaging [8].

In accordance with the general anthropometric trends, significant sexual dimorphism was noted with male dimensions larger than females. This has implications with the selection of prosthetic parts. In total shoulder arthroplasty, both the humeral stem and head should be sized appropriately. The marked difference in groove width between males (12.10 mm) and females (10.25 mm) suggests that the approach to consider anatomic landmarks during surgery might be a "one-size-fits-all" approach [9]. Furthermore, the study stated that the right humerus usually had a steeper medial wall angle than the left. This could be an adaptive response to mechanical loading as the right arm is dominant in the majority of the population. Increased use and muscular force from the biceps may stimulate osseous remodeling increasing the depth of the groove and steepness of the wall to maintain stability of the tendon [10]. The measurements obtained from CT scans in this study were very similar to those obtained from dry bones, which validates the use of CT as a reliable tool for preoperative assessment. In the case of proximal humerus fractures, it is important to know the position of the groove perfectly. During the plating of fractures the screws inserted through the lateral plate may inadvertently pierce the bicipital groove if the surgeon underestimates the depth of this groove and the tendon may be attributed and rupture [11]. The data provided here on average depths and widths can be used as a guideline to avoid such iatrogenic injuries. Moreover, the identification of the supratubercular ridge in almost 7% of the radiological sample is also of significance. This is an anatomical variant that projects into the groove and could be responsible for a bicep tendon impingement. Preoperative recognition of this ridge on CT scans can alert the surgeon to the possibility of need for tendon tenodesis or tenotomy instead of repair, since the presence of this ridge makes the chance of preserving the tendon less likely to be successful [12]. In arthroplasty of the shoulder, the bicipital groove is frequently used as a landmark for rotational alignment (retroversion). However, the variability in width and the presence of osteophytes (often seen in the older populations undergoing replacement) can make this landmark difficult to see. The wide range of widths that we observed in our study (from about 8 mm to over 15 mm) is a caution when considering the center of the groove as a universal reference point without consideration of individual patient anatomy [13]. Relying on the groove without reference to the transepicondylar axis might cause malrotation of the humeral component. When compared to studies on western populations, the dimensions in our cohort appear to be slightly smaller reinforcing the concept that skeletal morphometry is population specific [14]. For example, data of European samples often have mean groove depths of more than 5 mm. This discrepancy highlights the importance of using population-specific information to design orthopedic implants and instruments in order to ensure good fit and function [15]. There are some limitations to the study. The dry bone sample lacked associated age and sex records, which limited the demographic analysis of that particular group of people. Additionally, while well suited for viewing bone detail, CT is not able to visualize the soft tissue stabilizers (transverse humeral ligament) that also contribute to stability [16]. Future research using MRI may give a more global picture of the fibro-osseous tunnel.

## Conclusion:

We show that there is great morphological variability of the bicipital groove in terms of sex and side. The high degree of concordance between dry bone and CT measurements validates the use of preoperative CT to provide extensive surgical planning. These morphometric standards are part of a more accurate, anatomy-based approach to shoulder surgery.

## Figures and Tables

**Table 1:** Morphometric measurements of the bicipital groove in dry humeri (Group A)

**Parameter**	**Right (n=40) Mean ± SD**	**Left (n=40) Mean ± SD**	**p-value**
Length (mm)	83.10 ± 5.05	81.80 ± 5.18	0.245
Width (mm)	11.65 ± 1.75	10.81 ± 1.88	0.042*
Depth (mm)	4.85 ± 1.15	4.45 ± 1.22	0.134
Medial Wall Angle (°C)	58.4°C ± 6.2°C	54.1°C ± 5.8°C	0.003*
Significant at p < 0.05

**Table 2:** Radiological dimensions of the bicipital groove on CT scan (Group B)

**Parameter**	**Male (n=40) Mean ± SD**	**Female (n=40) Mean ± SD**	**p-value**
Width (mm)	12.10±1.65	10.25±1.45	<0.001*
Depth (mm)	5.12±1.05	4.15±0.95	<0.001*
Medial Wall Angle (°C)	57.2°C±5.9°C	52.8°C±6.1°C	0.001*
Supratubercular Ridge (%)	17.5% (7/40)	10.0% (4/40)	0.329

**Table 3:** Correlation between groove depth and medial wall angle (Combined Group A & B)

**Groove Type**	**Depth Range (mm)**	**Mean Medial Wall Angle (°C)**	**N**	**Pearson Correlation (r)**	**p-value**
Shallow	< 4.0	41.5°C±4.2°C	29	0.78	<0.001*
Medium	4.0 - 6.0	56.2°C±5.1°C	102		
Deep	> 6.0	65.8°C±6.5°C	29		
